# Transcriptomic Time-Series Analyses of Gene Expression Profile During Zygotic Embryo Development in *Picea mongolica*

**DOI:** 10.3389/fgene.2021.738649

**Published:** 2021-09-29

**Authors:** Jia Yan, Ha buer, Ya ping Wang, Gegen zhula, Yu´e Bai

**Affiliations:** ^1^ Institute of Forest Tree Genetic Breeding, Forestry College, Inner Mongolia Agricultural University, Hohhot, China; ^2^ Life Science of College, Inner Mongolia University, Hohhot, China

**Keywords:** *Picea mongolica*, embryogenesis, zygotic embryo, transcriptomics, WGCNA

## Abstract

Zygotic embryogenesis is a critical process during seed development in gymnosperms. However, knowledge on the genome-wide transcriptional activation that guides this process in conifers is limited, especially in *Picea mongolica*. This tree species is endemic to semiarid habitats of Inner Mongolia in China. To extend what is known about the molecular events underpinning its zygotic embryogenesis, comparative transcriptomic analyses of gene expression in zygotic embryos were performed by RNA sequencing in *P. mongolica*. Our results showed that most changes in transcript levels occurred in the early embryonic pattering determination and formation of mature embryos. Transcripts related to embryogenic competence, cell division pattern, hormones, and stress response genes were identified during embryogenesis. Auxin is essential for early embryo patterning and pre-cotyledon embryonic formation. However, ABA is a major regulator of embryo maturation. Moreover, we found that methylation-related gene expression is associated with activation of early-stage embryos, late embryogenesis abundant proteins, and storage/energy-related genes with late and mature embryos. Furthermore, network analysis revealed stage-specific and multistage gene expression clusters during embryogenesis. WOX, MYB, AP2, and HLH transcription factors seem more relevant to embryogenesis in different stages. Our results provide large-scale and comprehensive transcriptome data for embryo development in *P. mongolica*. These data will lay a foundation for the protection and utilization of *P. mongolica* resources.

## Introduction

Embryonic development is a critical reproductive phase of sexually reproducing plant species. Embryogenesis takes place from the one-cell zygote stage to a mature embryo in plant seeds. The key events and regulation pathways of reproductive development and embryogenesis have been widely studied in model angiosperms, such as *Arabidopsis thaliana* (Hofmann et al., 2019; Dresselhaus and Jürgens, 2021; Hao et al., 2021). However, the gene regulation mechanism associated with embryogenesis progression in gymnosperms is still limited (Trontin et al., 2016). Moreover, striking differences have been observed during conifer embryo development, which have a brief period of free nuclear divisions before cellularization of the embryo (Singh, 1978). Therefore, the molecular regulation mechanism must be different in conifers.

Recently, with the establishment of plant regeneration *via* the somatic embryogenesis pathway, molecular research on embryogenesis has received extensive attention in conifers (Llebrés et al., 2018; Rodrigues et al., 2018; Liu et al., 2019; Rodrigues et al., 2019). It has been reported that douglas-fir LEAFY COTYLEDON1 (PmLEC1) is an active transcription factor during zygotic and somatic embryogenesis (Vetrici et al., 2021). Thermospermine synthase (ACL5) and diamine oxidase (DAO) expression is needed for zygotic embryogenesis in Scots pine (Vuosku et al., 2019). PaWOX2 and PaWOX8/9 are expressed in both zygotic and somatic embryos during early embryo development in *P. abies* (Palovaara et al., 2010). Although these results provide additional basic knowledge for further improvement of somatic embryogenesis in conifers, knowledge concerning the regulation of embryo development is still limited.

*Picea mongolica* is a species native to Inner Mongolia of China. It has adapted to extreme and highly stressful environments and has great commercial and ecological value for the construction of urban green spaces in arid areas (Lorenz et al., 2012). The conventional propagation methods for *P. mongolica* are mainly through seeds and cuttings. However, the bad growth environment affects the development of seeds, while propagation through cuttings is hindered by rooting problems. In a previous study, we established an effective somatic embryo (SE) reproduction system, the best pathway to enhance *P. mongolica* yield (Yan et al., 2021). We found that embryos of pre-cotyledon embryos stage are the best explants for EC induction. Therefore, a better understanding of the regulation mechanism and key genes involved in embryogenesis is necessary to improve the efficiency of our SE protocols.

It is known that large genomes and large amounts of repetitive DNA in conifers have severely restricted efforts to produce a conifer reference genome and characterize expressed sequences. To this end, RNA-Seq is an essential tool and advanced approach for transcriptome-wide analysis of differential gene expression. Recently, reference transcriptomes of many conifers during the period of zygotic embryogenesis have been reported, including maritime pine, Scots pine (*Pinus sylvestris*), and *Pinus pinaster* (de Vega-Bartol et al., 2013a; Vuosku et al., 2015; Merino et al., 2016). Based on the results of these studies, the transcripts associated with carbohydrate metabolism, monosaccharide transport, and epigenetic regulation may serve as reliable molecular markers for early embryogenesis (Vuosku et al., 2015; Merino et al., 2016).

In the present study, to gain insight into the regulatory pathways associated with zygotic embryo development and reveal the transcriptomic activation mechanism during embryogenesis, we sequenced the transcriptome of zygotic embryo (ZE) over several developmental stages covering most embryogenesis stages in *P. mongolica*. Then, differentially expressed genes (DEGs) were identified during seed development. Many genes related to transcriptional regulation, signal transduction, and metabolic pathways were identified. Additionally, weighted gene co-expression network analysis (WGCNA) was performed to reveal key genes for specific time points in seed development. Our results provide data on molecular and biochemical events associated with ZE development, a foundation for improving the vegetative propagation of conifers *via* somatic embryogenesis in *P. mongolica*. Moreover, this work expands our understanding of the development of ZEs in conifers.

## Materials and Methods

### Plant Material

**FIGURE 1:**
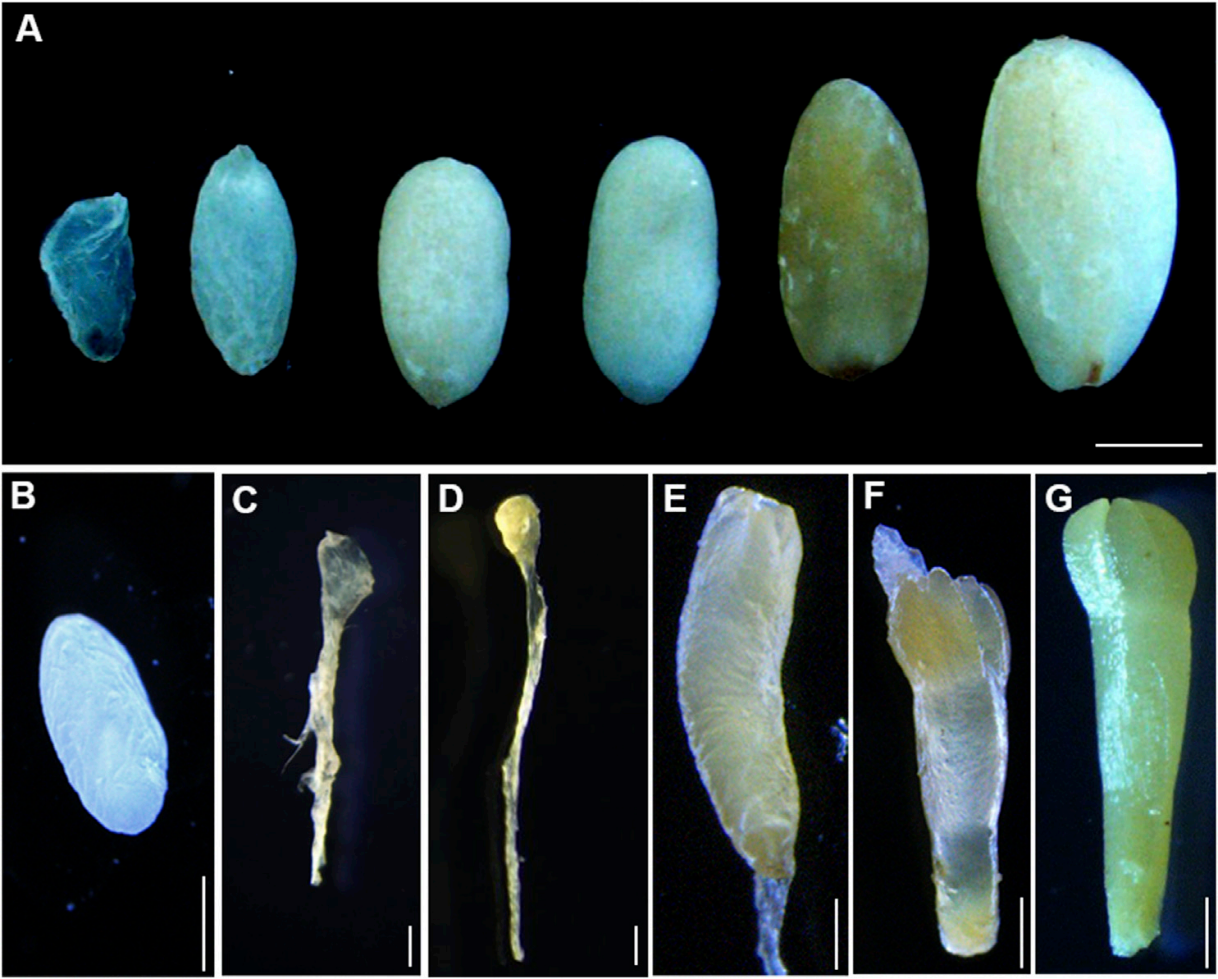
Development stages of immature zygotic embryos. **(A)** Developing seeds collected from open-pollinated trees on different dates after fertilization. Bars 1 mm. **(B**–**D)** Immature zygotic embryo after fertilization. **(B)** Megagametophy, **(C)** cleavage polyembryony, **(D)** dominant embryos, **(E)** pre-cotyledon embryos, **(F)** cotyledon embryos, and **(G)** mature embryos. Bars 0.1 mm.

Immature seeds of *P. mongolica* were obtained every 10 days after pollination between June and September 2019, from adult trees in the Baiyinaobao Nature Reserve of Inner Mongolia, China. Cones were periodically collected every week for all desired developmental stages of ZEs. The megagametophytes containing different developmental stages of ZEs were divided for RNA sequencing based on stereomicroscope observations (von Arnold et al., 2002), as follows: SDYS1 collected after 1 week of fertilization, which likely included early embryos in the proembryogenic stage, SDYS2 including the cleavage polyembryony in the early embryogenic stage, SDYS3 including the dominant embryos, SDYS4 including the pre-cotyledonary embryos, SDYS5 including the cotyledonary embryo, and SDYS6 including the mature embryo (Figure 1).

### RNA Sample Preparation

For transcriptome sequencing, total RNA was obtained using the Plant RNA kit (Sigma-Aldrich, St. Louis, MO, United States) following the manufacturer’s instructions. RNA degradation and contamination were detected in 1% agarose gels. RNA quantity and integrity were assessed using the NanoPhotometer spectrophotometer (NanoPhotometer purchased from Implen, Munich, Germany) and RNA Nano 6000 Assay Kit of the Bioanalyzer 2100 system (Agilent Technologies, CA, United States). Total RNA (1 µg) was used to generate sequencing libraries using the NEBNext^®^ Ultra™ RNA Library Prep Kit for Illumina (NEB, Ipswich, MA, United States) sequencing.

### DEG Statistics

Differential expression analysis was performed using the DESeq2 R package based on all obtained reads per kilo base per million mapped reads (RPKM) (1.16.1) (Love et al., 2014). Pairwise differential expression analysis was conducted among six group samples in sequence. Each set of samples contains three replicates. Transcripts that have log2FC ≥ 1 or log2FC ≤ 1 and *p*-value < 0.05 were used to determine DEGs. Further analysis will be based on the fold-change value of EEGs using Expander 7 software (Ulitsky et al., 2010) with the K-means algorithm (Shamir et al., 2005).

### Functional Annotation and GO Analysis

The agriGO (version 2.0) (http://systemsbiology.cau.edu.cn/agriGOv2/) was used in Gene Ontology (GO) enrichment analysis of obtained DEGs, which was corrected by a *p* value less than 0.05. The cluster Profiler R package was used to test the enrichment of DEGs in KEGG pathways. Venn diagrams were constructed using online software (http://bioinformatics.psb.ugent.be/webtools/Venn/). Hierarchical clustering heat maps were built, and cluster analyses were conducted using MultiExperiment Viewer (http://www2.heatmapper.ca/expression/).

### Gene Co-Expression Network

The R package WGCNA V1.41-1 was used for WGCNA analysis (Langfelder and Horvath, 2008). After filtering, the automatic network construction function blockwiseModules was used to construct co-expression modules based on gene expression values. Cluster correlation was used to construct cluster families within the network. The network image was created using Cytoscape software.

### Quantitative Real-Time PCR

Total RNA was obtained from each sample using an RNA extraction kit (Takara, Japan), and cDNA was obtained using the PrimeScript™ RT Reagent Kit (Takara, Japan). PCRs were performed using AceQ qPCR SYBR Green Master Mix with the following thermal cycling conditions: 95°C for 10  min, 40 cycles of 95°C for 10 s, and 60°C for 60  s (de Vega-Bartol et al., 2013b). Relative gene expression was quantified using the 2−ΔΔCt method (Cao et al., 2016).

## Results

### Dynamics of Gene Expression During the Development of ZE

**FIGURE 2:**
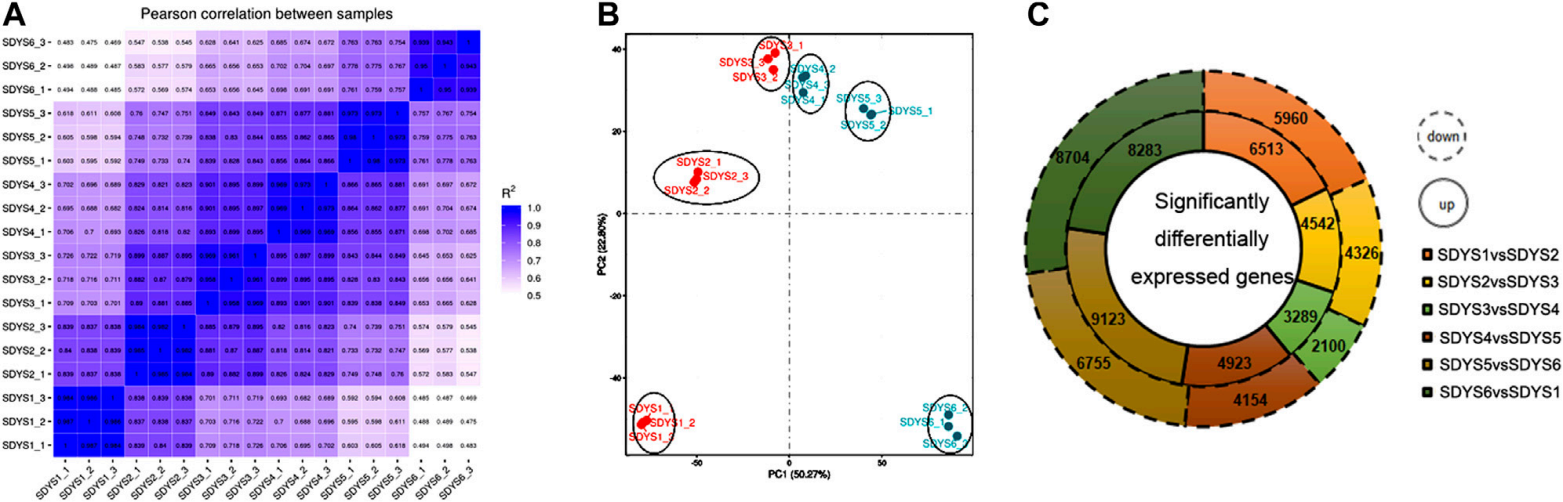
Transcription during embryonic development of *P. mongolica*. **(A)** Correlation analysis among all samples. **(B)** Principal component analysis of the transcriptomes of samples from six stages. **(C)** Differentially expressed genes in each stage.

To evaluate the molecular mechanisms governing the embryogenesis of *P. mongolica*, we isolated ZEs from June to September after pollination (Figure 1). Gene expression profiles were developed using RNA-seq as described in the methods section. After trimming adapter sequences and removing low-quality and multi-matched reads, more than 136.7 million unique clean reads and 20.5- to 30.4-G clean bases remained. A total of 76–80% clean reads were mapped to the reference genome of the *Norway spruce*. All samples were highly correlated and therefore clustered together (Figure 2A). Moreover, a principal component analysis (PCA) result demonstrated that the three replicates of each developmental stages can be clearly distinguished (Figure 2B).

**FIGURE 3:**
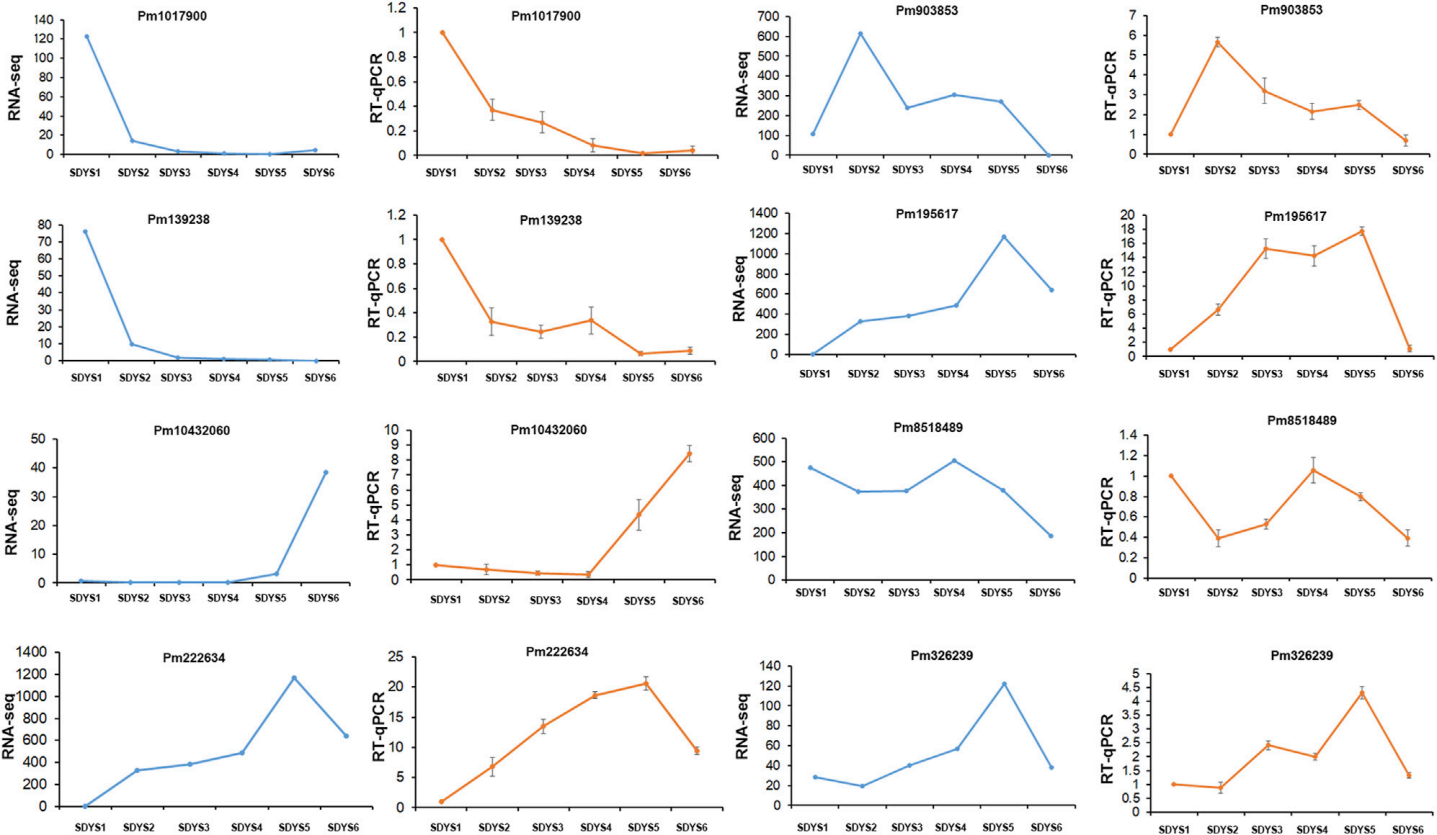
Validation of RNA-seq results *via* RT-qPCR Fold changes for selected transcripts obtained by RNA-seq and RT-qPCR are shown for each developmental time point. Transcript levels are means ± SD of three biological replicates.

We further analyzed DEGs through pairwise comparisons between consecutive stages of development. A two-fold difference (log2FC ≥ 1 or ≤ −1) and false discovery rate of 0.05 or less were used as thresholds. Each pairwise comparison identified a large proportion of DEGs, including 12,473 in proembryogenic versus cleavage polyembryony stages, 8,868 in cleavage polyembryony versus dominant embryo stages, 5,389 in dominant embryos versus pre-cotyledonary embryos, 9,077 in pre-cotyledonary embryos versus cotyledonary embryos, 15,878 in cotyledonary embryos versus mature embryos, and 16,987 in mature embryos versus proembryo embryos (Figure 2C). Next, we selected eight transcripts as candidate genes for further investigation of their expression patterns using RT-qPCR to evaluate their transcriptome data. The expression of these genes was consistent with the transcriptome data (Figure 3).

**FIGURE 4:**
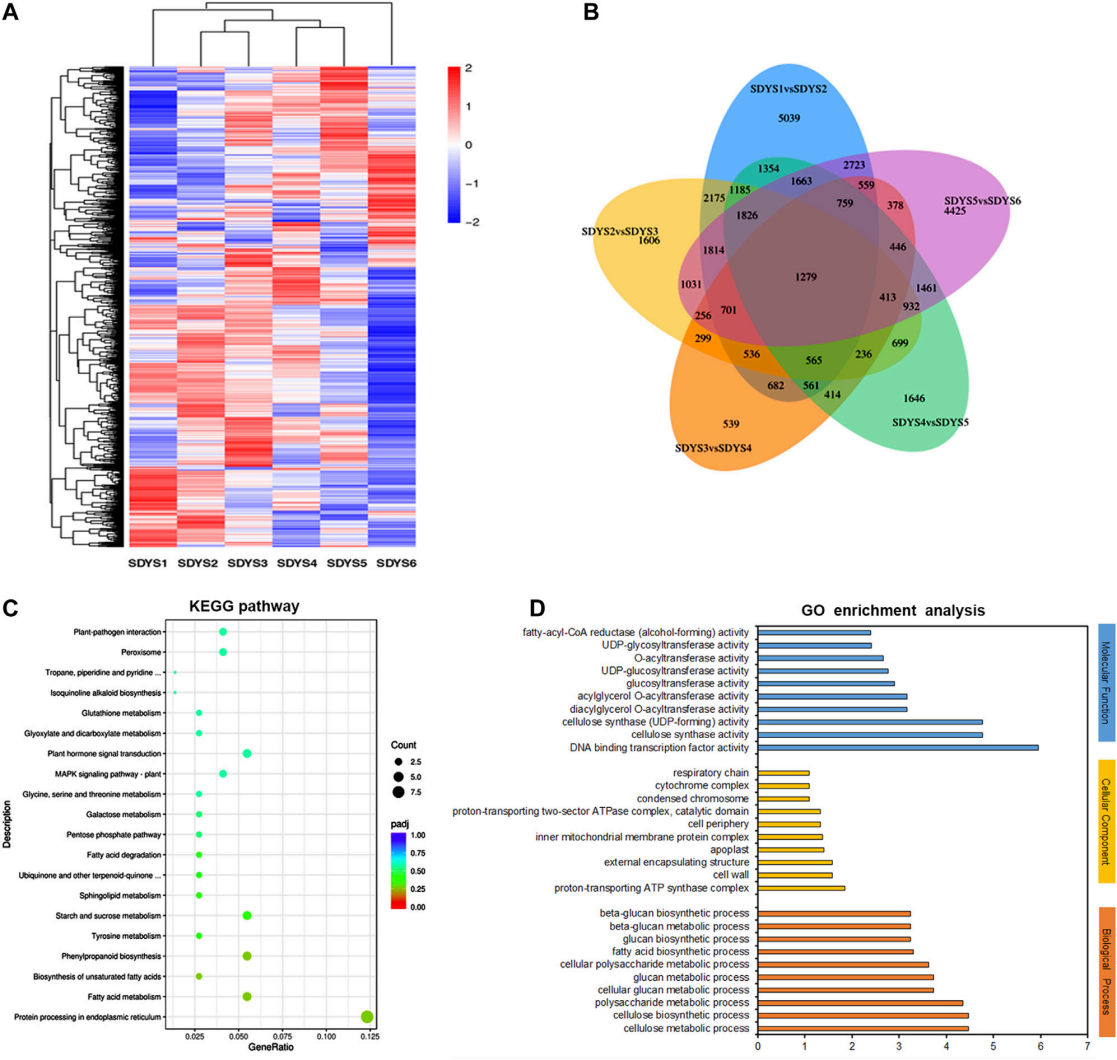
Identification and analysis of differentially expressed genes during embryonic development stages. **(A)** Heat map, **(B)** Venn diagram, **(C)** KEGG annotation, and **(D)** GO enrichment analysis of differentially expressed genes in six embryonic development stages.

Based on the significantly expressed genes, a heat map was generated after background subtraction and normalization to represent the global dynamics of gene expression during zygotic embryogenesis in *P. mongolica* (Figure 4A). Furthermore, Venn diagram analysis showed that 5,039 transcripts were differentially expressed from stages 1 to 2, 1,606 from stages 2 to 3, 639 DEGs from stages 3 to 4, 1,646 from stages 4 to 5, and 4,425 from stages 5 to 6. Additionally, 1,279 genes appeared in five comparisons across all six stages of embryo development (Figure 4B). In all, the greatest difference in the number of DEGs was in the early embryogenic stage and later to mature embryonic stage. In contrary, little difference was observed between stages 3 and 4. Based on these results, embryonic developmental stages can be divided into the following: stage 1 is the proembryogenic phase; stage 2 is the early embryogenic phase; stages 3–4 are the mid-embryogenic phase; stage 5 is the latter embryogenic phase, and stage 6 is the mature embryo in *P. mongolica*.

### Functional Regulation of DEGs During the Development of ZE

Furthermore, we classified these DEGs based on KEGG pathways, which were mainly enriched in protein processing of the endoplasmic reticulum, fatty acid metabolism, starch and sucrose metabolism, phenylpropanoid biosynthesis, plant hormone signal transduction, and MAPK signaling pathway (Figure 4C). Subsequently, GO enrichment analysis was performed to further characterize their biological roles. Most biological process (BP) terms were enriched in cellulose metabolic and biosynthetic processes, polysaccharide metabolic processes, and glucan metabolic processes. In the molecular function (MF) category, DNA-binding transcription factor activity and cellulose synthase activity were significantly enriched. The significantly enriched cellular component (CC) terms were cell wall and apoplast (Figure 4D). In brief, many DEGs were involved in the regulation of embryonic development, and these pathways have great significance for further revealing the molecular events involved in zygotic embryogenesis in *P. mongolica*.

### DEG Identification Across Whole-Embryo Developmental Stages

**FIGURE 5:**
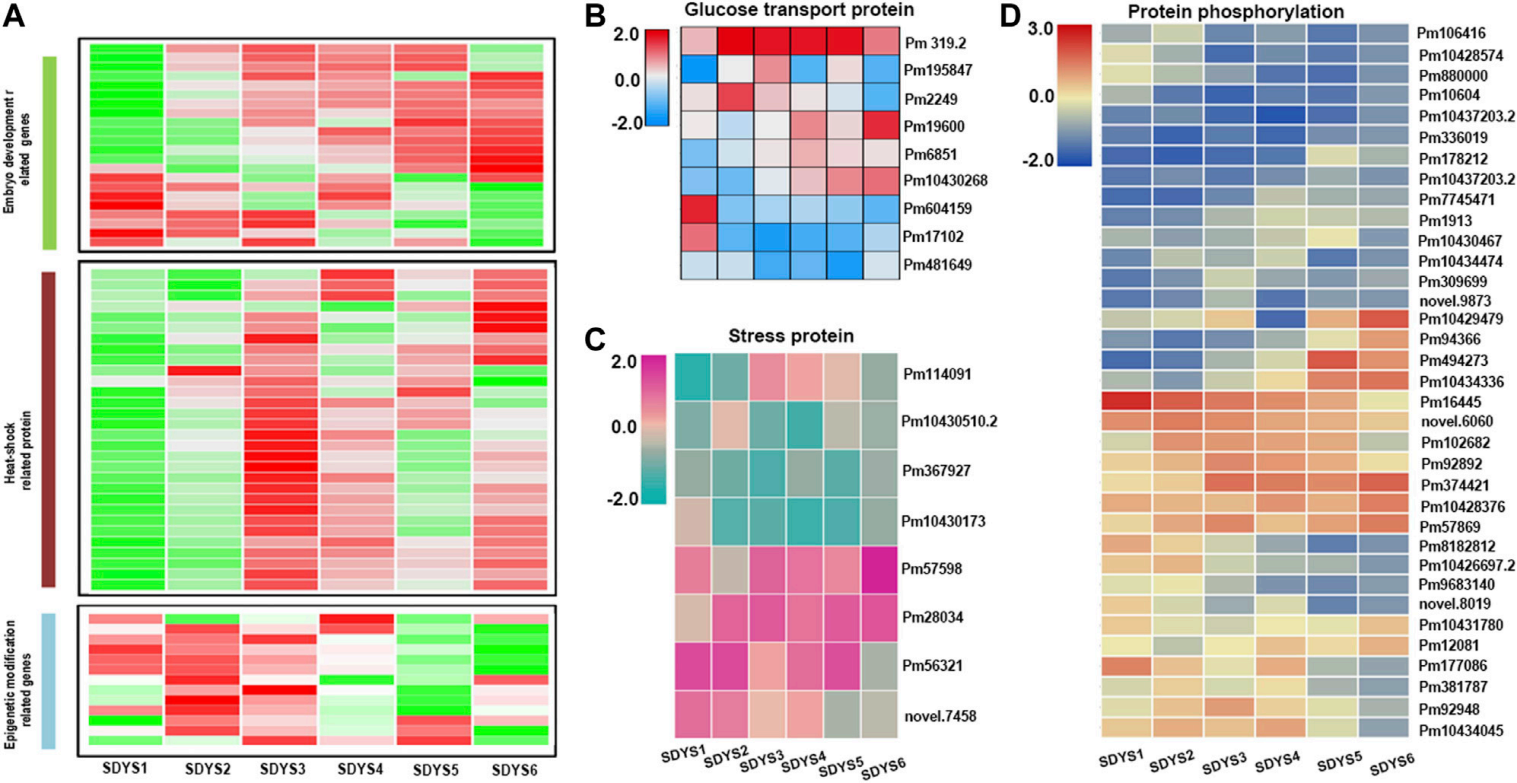
Clustering of differentially expressed genes identified in five comparisons across all six stages of embryo development. **(A)** Heat map of differentially expressed genes. **(B)** Glucose transport genes, **(C)** stress response genes, and **(D)** protein kinases and phosphatases.

Among the DEGs identified, 1,279 genes appeared in five comparisons across all six stages of embryo development. Based on functional annotations and accumulated data on model plant systems, statistical results showed that the expressions of all six LEA genes, seven seed storage proteins, three seed maturation protein-encoding genes, and one seed dormancy gene were different in every developmental stage (Figure 5A). These associated genes were enriched in post-embryonic development, seed development, and fruit development (Supplementary Figure S1). In addition, many epigenetic regulation components were found during embryogenesis. Several putative histone subunit homologs and methyltransferases had different expression profiles across developmental stages. Additionally, genes associated with glucose transport and stress protein were also differentially expressed (Figures 5B–D). Five sugar efflux transporters and three sugar transporter genes involved in glucose metabolism and the transport pathway were expressed during ZE maturation. Moreover, stress response genes and HSP family genes were obviously differentially expressed in all six developmental stages during ZE maturation (Supplementary Figure S1). Importantly, several tyrosine phosphatases and PP2C were downregulated in later embryogenic stages, suggesting that the ABA signal transduction cascade plays an important role in later embryogenesis.

### Specific Expression of DEGs During Each Embryogenesis Phase

**FIGURE 6:**
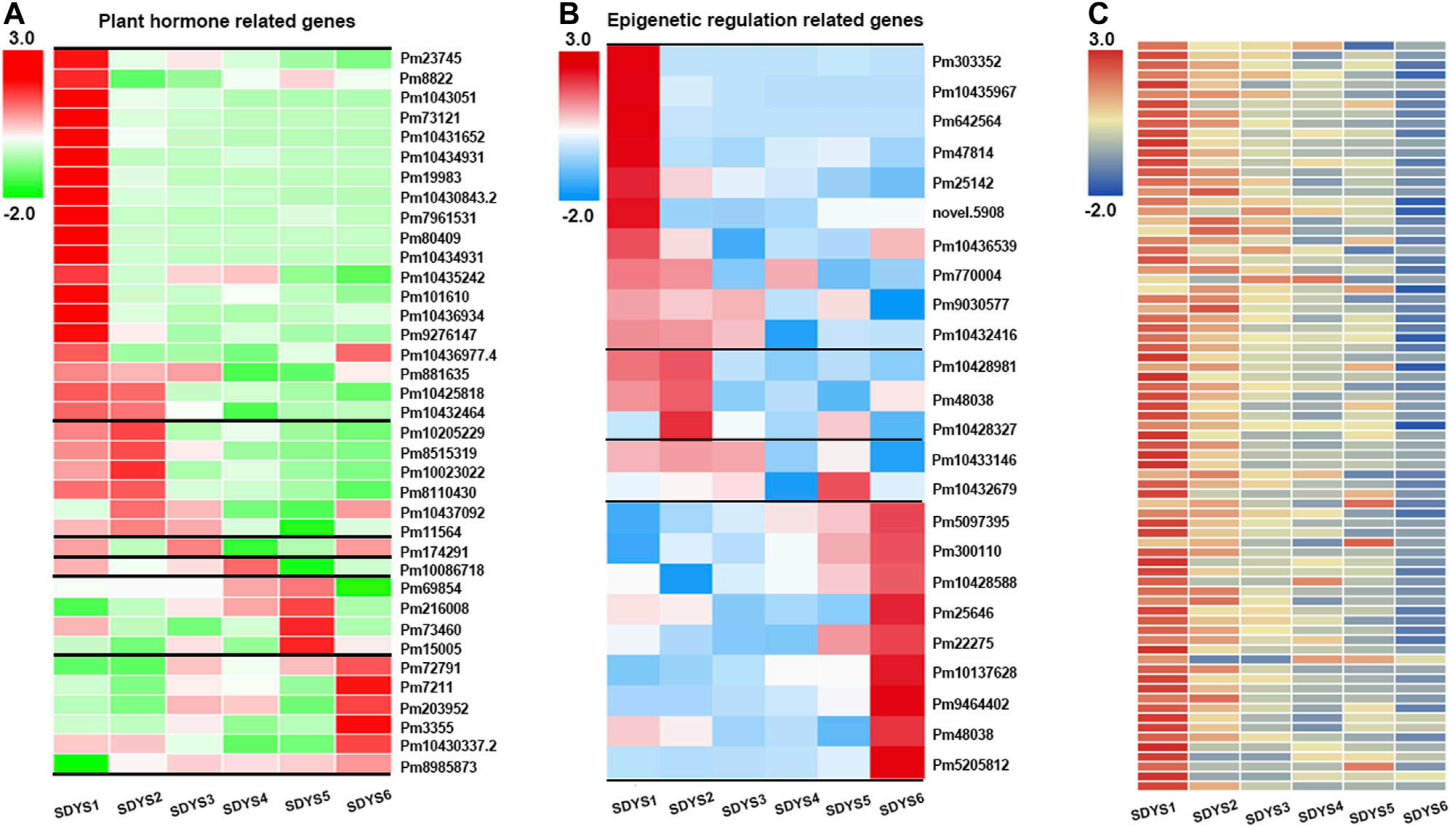
Heat map of specific expression of differentially expressed genes identified during each embryogenesis phase. **(A)** Plant hormone-related gene, **(B)** epigenetic regulation-related gene, and **(C)** NB-ARC-related gene expression in six different developmental stages.

Because multiple phytohormone-related genes are involved in the acquisition of embryogenic competence, we further analyzed the transcriptome data to gain insights into the function of plant hormones in zygotic embryogenesis (Figure 6A). In the early embryogenic stage, auxin responsive genes, auxin transport genes AUX/IAA, and gibberellin response genes were upregulated in early embryogenesis. Conversely, ethylene-responsive protein kinase Le-CTR1 and ABA-related protein PP2C phosphatase were downregulated in this stage. In addition, several PP2C was still upregulated in early to middle embryogenesis. EIN3, which could initiate downstream transcriptional cascades for ethylene responses, was upregulated in the pre-cotyledon embryo stage. Auxin transporter including AUX/IAA and PIN genes and auxin responsive genes were upregulated in the cotyledon embryo stage. Subsequent seed maturation, PP2C phosphatase, and AUX/IAA family genes were significantly increased. Taken together, these results suggested that auxin, GA, and ethylene may participate in early embryonic initiation and cotyledon embryonic formation. Auxin and ABA synergistically regulate late embryonic development and seed maturation in *P. mongolica*.

Furthermore, transcripts related to epigenetic regulation were also found in different developmental phases (Figure 6B). SAM-dependent carboxyl methyltransferase was found during early embryogenesis, suggesting that DNA methylation has an important role in embryonic development of *P. mongolica.* In addition, chromatin modification-related genes, such as Jumonji C-domain histone demethylase-encoding gene, showed maximum expression during early embryogenesis. The expression of three transcripts related to histone deacetylase, which is usually associated with transposon silencing, were also increased in early embryogenesis. Therefore, histone methylation and acetylation modification play a relevant role in the early embryogenic phase of *P. mongolica*.

Most changes in transcript levels occurred in the early embryonic pattering determination and mature embryos formation. DEGs between stage 6 and stage 1 were identified to further investigate transcriptional regulation in ZE development. In the megagametophytes including proembryos and auxin polar transport (AUX/IAA and PIN3), response TFs (MYB, AP2, bZIP, and B3) were highly abundant. More upregulated transcripts were highly enriched in response to stimulus, such as salt, drought, cold, and wound stress. Moreover, histone H2A, lysine-specific demethylase JMJ706 and JMJ25, and histone RNA stem-loop-binding two genes were up-expressed in early proembryos. On the contrary, histone acetylation-related genes (HAC1 and histone deacetylase 6) and chromatin formation or remodeling genes (MBD9, RRC1, and SNF2-related transcripts) were highly expressed at the mature phases. ABA response, dehydration, photosynthesis initiation, and storage/energy-related genes were associated with mature stages of embryo development. In addition, many DEGs were annotated as containing “terminal inverse repeats (TIR),” “leucine rich repeats,” or “NB-ARC domains,” which are molecular switches implicated in immune signal transduction mechanisms (Figure 6C). These genes were highly abundant at stage 1 but declined successively during later developmental stages. These results are consistent with the previous report of transcriptome in maritime pine (Gonçalves et al., 2005; Rodrigues et al., 2018).

### Stage-Specific Gene Co-Expression Networks *via* WGCNA

**FIGURE 7:**
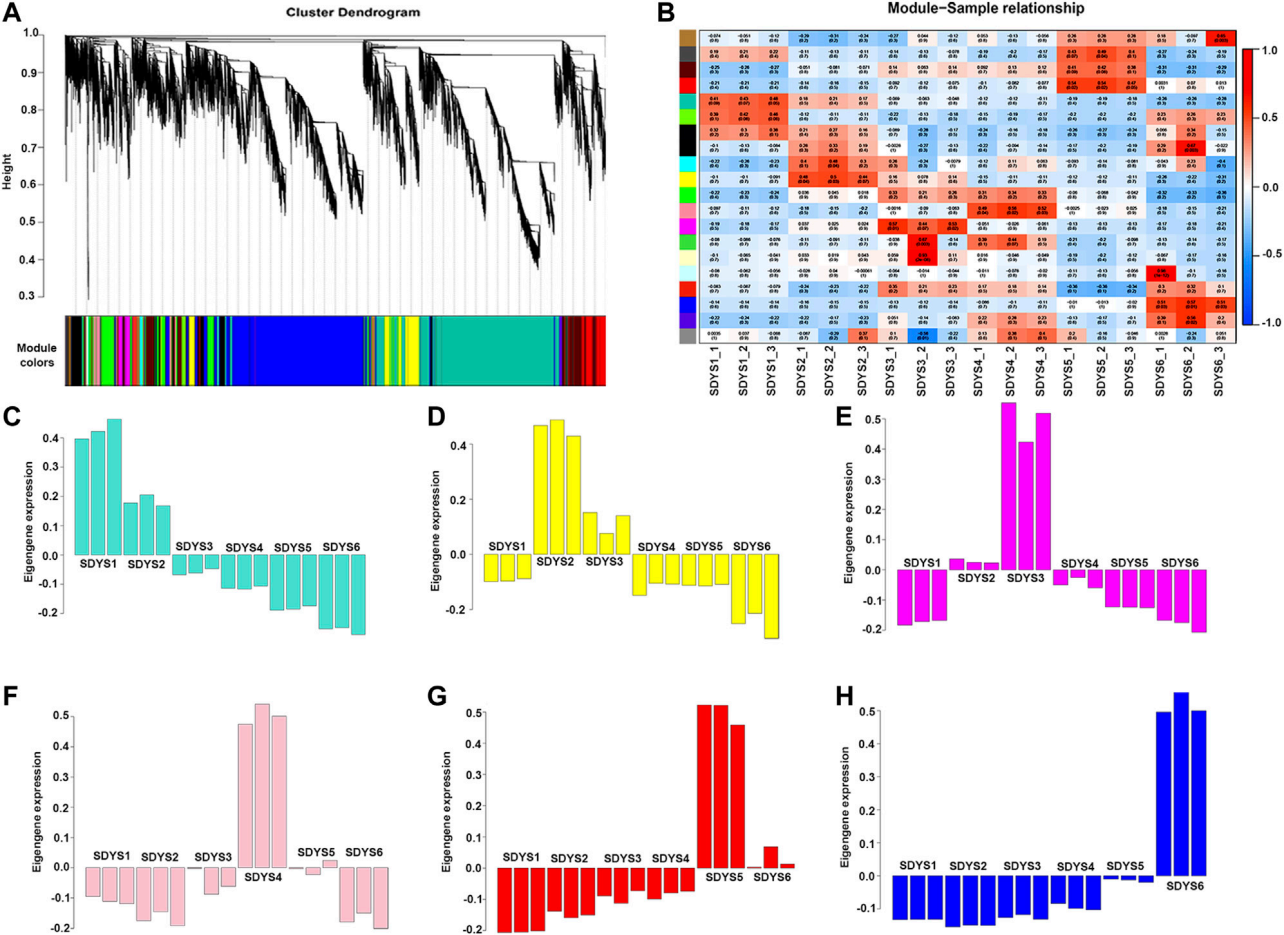
Zygotic embryo development related module performed by WGCNA. **(A)** Cluster dendrogram of co-expressed genes. **(B)** Heat map of correlation between co-expression modules. **(C**–**H)** Correlation between co-expression modules and traits. A negative value represents a negative correlation, and a positive value represents a positive correlation.

Because all DEGs represent different functions, a WGCNA assay was used to define clusters and obtain gene modules with specific patterns of expression during the six-embryo development stages (Figure 7). Based on expression pattern and tissue specificity, 20 gene modules were identified. Six stage-specific modules were significantly correlated with one specific stage of embryonic development (r > 0.8 and *p* value < 0.001). Next, we analyzed the functions of these genes in each model and found that the translation initiation factor, CCAAT-box binding factor, and mRNA splicing factor were significantly associated with early embryogenic stage, suggesting that this period may be the transcriptional activation stage. Heat shock protein was significantly correlated with dominant embryonic developmental stage. Auxin-responsive protein was mainly enriched in the pre-cotyledon stage. Several late-embryogenesis abundant-like proteins were found during late embryogenesis and embryonic maturation stages. Therefore, LEA are likely key genes for embryo maturation in *P. mongolica*. In addition, the top 50 genes associated with MEdarkred were of unknown function. This may be due to incomplete genome annotation in *P. mongolica.*

### Identification of Transcripts for Embryo-Specific Markers

**FIGURE 8:**
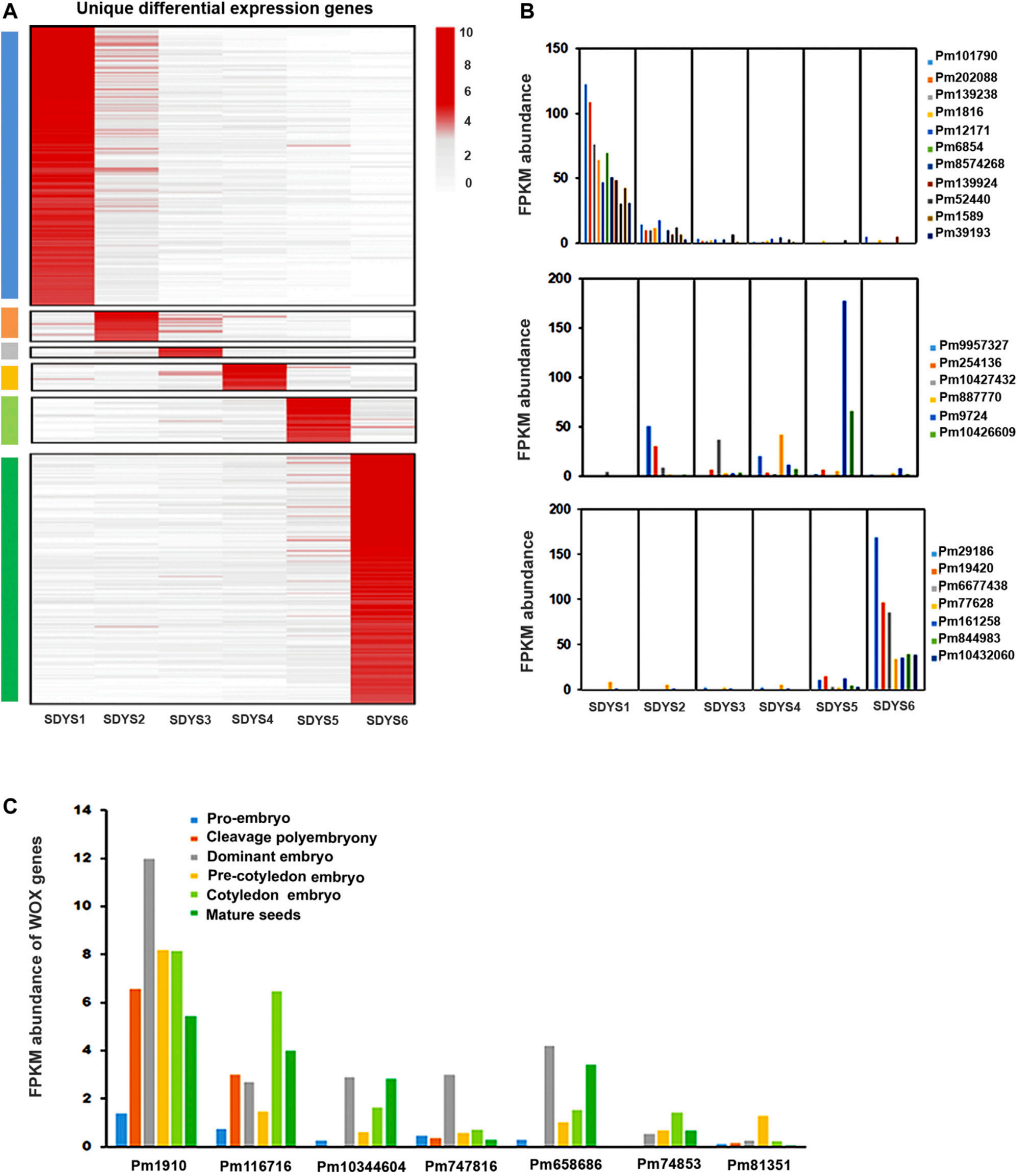
Functions of embryonic development-specific differentially expressed genes. **(A)** Hierarchical clustering of fold changes in transcript abundance for all specific DEGs in each embryonic developmental stage. **(B**,**C)** Expression of TFs and WOX genes in different embryonic development stages.

To further identify embryonic markers in *P. mongolica*, the MGFR tool (El Amrani et al., 2015) was used to analyze the DEGs of all samples. Fragments per kilobase of transcript per million mapped reads > 30 in at least one embryonic stage were used as the standard for DEG evaluation. We identified 184 transcripts in pro-embryos, 20 in early embryos, 7 in dominant embryos, 18 in pre-cotyledon embryo, 29 in cotyledon embryos, and 164 in mature seed embryos. These transcripts seem to be candidate genes for embryo-specific markers (Figure 8A). Considering that TFs are major determinants of embryogenesis, they are best for use as embryonic markers. We investigated transcripts likely TFs, whose levels were at least fourfold higher than other transcripts in a set of phase-enriched markers. There were MYB, GATA, TCP, and AP2 TFs associated with pro-embryo initiation. MYB was a key TF family in early embryo formation; HLH and AP2 TFs were highly expressed in the embryonic maturation stage (Figure 8B).

In addition, we also identified the known transcription factor marker gene WOX during embryonic morphogenesis (Figure 8C). Pm1910, Pm116716, and Pm10344604, likely putative orthologs of WOX8, were found to be downregulated in early embryogenesis. Pm10344604, a putative ortholog of WOX11, was upregulated in the dominant embryonic stage. Pm658686 was likely WOX12 and showed high expression in this stage. Pm81351 (a putative ortholog of WOX8) was strongly induced in the pre-cotyledonary stage. Pm74653 (WOX5) was upregulated in the cotyledonary stage.

### Expression Profiles of Transcription Factor Genes in Different Developmental Stages

**FIGURE 9:**
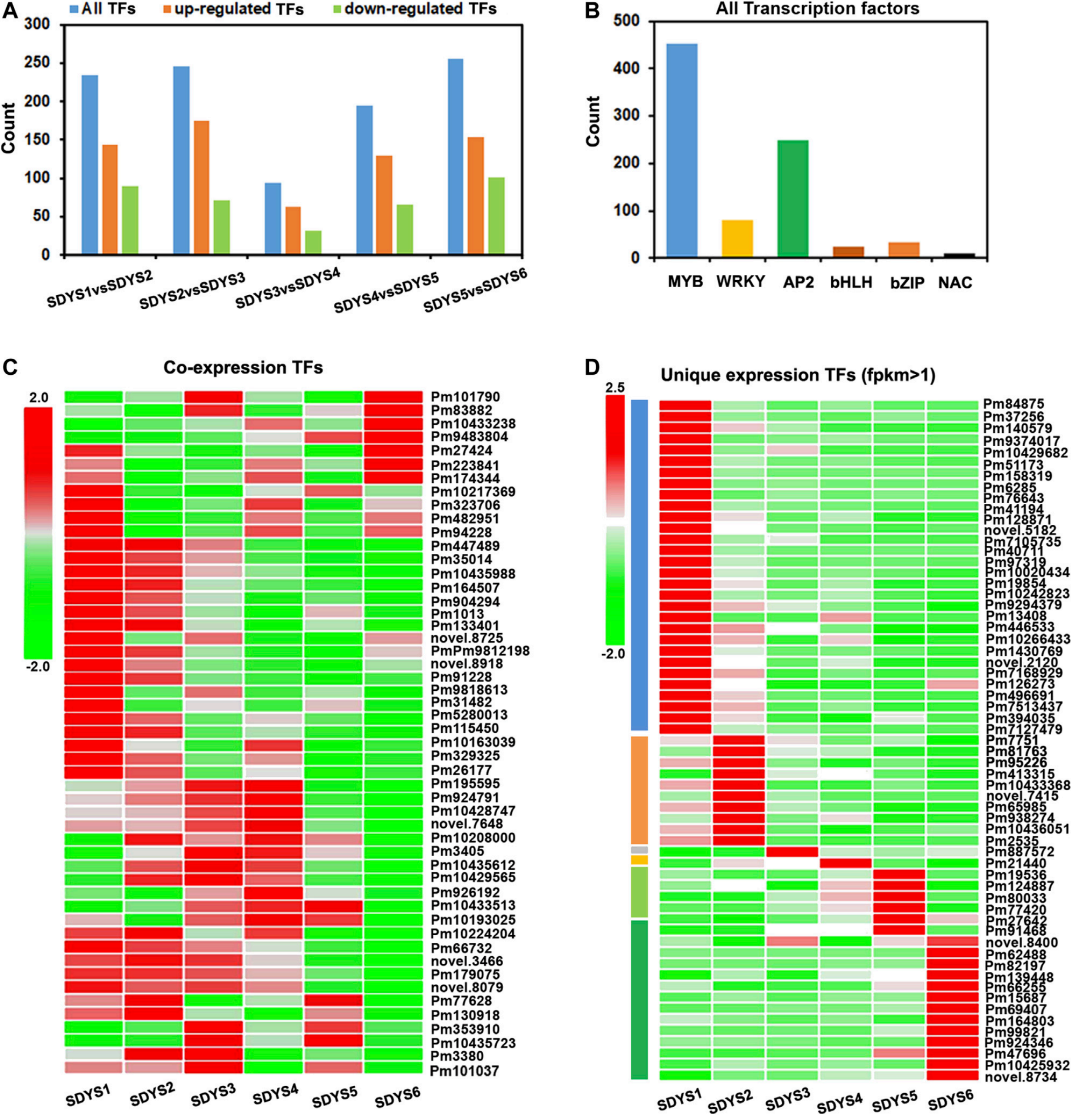
Differentially expressed TFs related to embryo development of *P. mongolica*. **(A**,**B)** All TFs identified in pairwise comparisons. **(C)** Heat map of TFs from 1,279 DEGs shared in all six groups. **(D)** Heat map of TFs from each embryo development-specific DEG.

TFs play a significant role in cell fate determination during embryogenesis. Hence, we further identified TFs involved in embryonic development in *P. mongolica* based on model plant systems (Figure 9). In all TFs, 144 were specifically upregulated and 90 were downregulated from pro-embryo to early embryo formation; 175 TFs were specifically upregulated and 71 were downregulated from early embryo to dominant embryo generation; 94 TFs (62 upregulated and 32 downregulated) in dominant embryo stages compared with pre-cotyledon embryonic stages; and 129 TFs were upregulated and 66 downregulated in the cotyledon embryonic period. One hundred fifty-four were upregulated and 101 downregulated from the cotyledon embryonic stage to the mature seeds (Figures 9A,C). In addition, we further analyzed the types of transcription factors in all DEGs (Figure 9B). They are mainly enriched in MYB, AP2, WRKY, HLH, bZIP, and NAC TF families. Furthermore, we analyzed the TFs in differentially expressed transcripts shared by the six groups (Figure 9D). We found that MYB TFs were upregulated in early embryogenesis. A putative WRKY transcript was strongly upregulated in middle embryogenesis. The AP2 and MYB transcripts were crucial in the transition from early embryogenesis to the pre-cotyledonary stage. Additionally, GATA, TCP, BES1/BZR1, and WRKY TFs were upregulated in mature embryos.

## Discussion

Many genes are required for embryonic development. For example, 289 genes have been reported to be embryo-specific genes in *A. thaliana*. In this study, RNA-seq technology was used to provide a comprehensive overview of the transcriptome of developing embryo in *P. mongolica*. We focused on the regulation of gene expression over the entire developmental process. In here, plant embryogenesis related the plant hormone pathways, stress response, epigenetic control, protein phosphorylation, and transcription factor regulation patterns which were fully interpreted at the transcription level in *P. mongolica*.

### Plant Hormone Pathways in ZE Development

Auxin plays a considerable role in cellular patterning during embryogenesis. Its biosynthesis and polar transport-related localization are crucial in plant embryogenesis (Pérez-Pastrana et al., 2019). A previous study found that auxin-induced genes, such as ARF16, significantly increase in early cotyledonary embryonic stages in *P. pinaster* (de Vega-Bartol et al., 2013a). ARF16 represses WOX5 transcription, which is required to maintain pluripotent *columella* stem cells (Radoeva et al., 2019). In our study, we found that the auxin transport carrier genes AUX and PIN3 were upregulated in early embryonic stages. Moreover, its expression was also increased from the early cotyledonary to later embryonic stages. Thus, these results suggest that auxin biosynthesis, auxin transport, and auxin response are more prominent in early embryogenesis and affect cotyledonary embryonic pattern formation. This result is consistent with a previous report that auxin influx (AUX1, LAX1, and LAX2) is needed for cellular patterning in early embryogenesis in *Arabidopsis* (Robert et al., 2015). In addition, we also found that WOX8 and several auxin response TFs were specifically expressed in early embryogenesis. These findings provide a framework for processes that the transportation and accumulation of auxin likely activate the expression of these TFs during early embryogenesis in *P. mongolica.*

In addition, abscisic acid (ABA) plays a critical role in maintaining embryogenesis by regulating tissue differentiation and determining the physiological characteristics of late embryos (Pérez et al., 2015). Moreover, protein accumulation is generally under the control of ABA in conifer (Högberg et al., 2001). In this study, we statistically analyzed the expression of PP2C, an ABA signal transducer and key factor protein phosphatase in different ZE developmental stages. Six transcripts annotated to PP2C phosphatase were downregulated in the cotyledonary embryonic stage. Moreover, genes associated with ABA response and dehydration are more highly expressed at the embryonic mature phases. These results suggest that ABA play a critical role in the embryonic maturation processes of *P. mongolica*.

### Epigenetic Regulation in ZE Development

Epigenetic reprogramming, such as histone posttranslational modifications, DNA methylation, and chromatin-remodeling maintenance, has co-regulated functions during early embryogenesis in pine (de Vega-Bartol et al., 2013b; Trontin et al., 2016). HAD2, an acetylation regulation gene, is particularly increased in the early cotyledonary stage in *P. pinaster* (Rodrigues et al., 2018). We found that histone H2A histone subunits and lysine-specific demethylases show a higher abundance in early embryogenesis. Thus, histone posttranslational modifications might drive the expression of the transcriptome of early embryos in *P. mongolica*. Furthermore, histone acetylation-related genes and chromatin formation or remodeling genes were found in early embryogenesis. Similar results have been reported during maritime pine and *Norway spruce* embryogenesis (Uddenberg et al., 2011; Rodrigues et al., 2018). In addition, our results showed that several SAM-dependent carboxyl methyltransferase and other methyltransferases show different expression profiles. In general, DNA methylation is important for embryogenesis in *P. mongolica*.

### Transcriptional Regulation During the Development of the ZE

A large number of TFs have been reported to regulate cell fate differentiation events, which determine the first zygotic division during embryogenesis. For example, the MYB TF family is expressed in early seed development (Avilés-Viñas et al., 2021). Moreover, MYB interacts with bHLH to regulate seed maturation. Network analysis showed that these TFs also associated with FUS3 and WOX12 in seed maturation (Tian et al., 2020). In here, we focused on TFs associated with plant development, phytohormones, and stress responses. The plant development-related MYB, AP2, and bZIP TF families were upregulated during early ZE development. The expressions of LEA, HSP, and HLH genes were increased during late ZE development. Additionally, homologues of the PLATZ and SRF family of plant-specific TFs, whose roles are poorly known, were also observed.

WOX genes (WOX2, WOX8, WOX1, and WOX3) play an important role during the ZE development. WOX2 is expressed in the apical daughter cell, and WOX8 is specifically expressed in the basal daughter cells of the zygote (Sakakibara et al., 2014; Armenta-Medina et al., 2021; Avilés-Viñas et al., 2021). The YDA-MAP signaling cascade phosphorylates and activates WRKY2 to promote WOX8 transcription during zygote development (Ueda et al., 2011; Horstman et al., 2017). We also found that WOX8 was specifically expressed in the early embryogenic stage, WOX11/12 were specifically expressed in the dominant embryonic stage, and WOX5 was upregulated in the cotyledonary stage. Additionally, a homolog of the WRKY family was also upregulated during this period. This may be consistent with regulation patterns in *Arabidopsis* that auxin response leads to activation of WRKY2 and expression WOX during embryogenesis.

### Genes That Are Sufficient for Embryogenesis in *P. mongolica*

Several genes are sufficient for embryogenesis when ectopically expressed. For example, when LEC1 and LEC2 are expressed in seedlings, SE is induced (Osorio-Montalvo et al., 2020). SERK1 encodes a leucine-rich repeat (LRR) transmembrane RLK, which induces SE when ectopically expressed. In addition, several TFs, such as AGAMOUS-Like15 (AGL15) and AGAMOUS-Like18 (AGL18), may also induce SE development (Zheng et al., 2016; Wójcik et al., 2020). The APETALA2 (AP2) domain TFs BABY BOOM/PLETHORA4 (BBM/PLT4), PLETHORA2 (PLT2), and WOUND INDUCED DEDIFFERENTIATION (WIND) promote SE in seedlings (DuBuc et al., 2020). Our transcriptome profiling data represent a valuable foundation for identifying genes, which are specifically expressed in particular stages of embryogenesis. We established a set of stringently defined temporal markers for embryogenesis. Transcription factors, including MYB, AP2, GATA, and TCP, could be markers during morphogenesis in early embryonic stages. AP2 and bHLH transcription factors are markers in the later embryonic stages to seed maturation. Heat shock proteins are specifically expressed in the domain embryo stage and serval auxin response genes in the pre-cotyledon embryo stage; late-embryogenesis abundant proteins are abundant during later embryogenesis. Therefore, these genes may be sufficient for embryogenesis and SE formation when ectopically expressed in *P. mongolica.*

In summary, our results establish a set of stringently defined temporal markers for identifying the key players involved in embryogenesis of *P. mongolica*. Our datasets and approach are expected to facilitate the discovery of molecular mechanisms underlying patterns of embryonic development. Moreover, functional understanding of these shared and distinct expression patterns of signaling, transcriptional, and epigenetic factors will help to address how embryonic development shapes the divergence of seed development in *P. mongolica.* Furthermore, our data lay a foundation for the protection and utilization of *P. mongolica* resources.

## Data Availability

The datasets presented in this study can be found in online repositories. The names of the repository/repositories and accession number(s) can be found below: SRA: SRR12338997, PRJNA649217.
